# Mobile health (mHealth)-supported community management of COPD: a prospective real-world study on clinical outcomes and exacerbation reduction

**DOI:** 10.3389/fpubh.2026.1836687

**Published:** 2026-05-07

**Authors:** Xi'ou Yang, Yonghua Zhang, Haihua Hong, Jianghong He, Yinan Yao, Yongbin Chen

**Affiliations:** 1 Beilun Branch of the First Affiliated Hospital of Medical College of Zhejiang University, Ningbo, Zhejiang, China; 2 Department of Infectious Diseases, People’s Hospital of Beilun District, Ningbo, Zhejiang, China; 3 Department of Respiratory Medicine, People’s Hospital of Beilun District, Ningbo, Zhejiang, China; 4 Department of Respiratory Medicine, The First Affiliated Hospital of Zhejiang University School of Medicine, Hangzhou, Zhejiang, China

**Keywords:** chronic obstructive pulmonary disease (COPD), community-based management, hierarchical diagnosis and treatment, mHealth, pulmonary function

## Abstract

**Background:**

Chronic obstructive pulmonary disease (COPD) remains a major public health challenge in China, with high prevalence and suboptimal rates of standardized long-term management. Conventional community-based management models are often limited by poor continuity and accessibility. Mobile health (mHealth) technologies offer a promising approach to enhance chronic disease management. This study aimed to evaluate the feasibility and real-world effectiveness of an mHealth-supported COPD management system in improving clinical outcomes and reducing exacerbations.

**Methods:**

An mHealth-based COPD management platform integrated into the WeChat ecosystem was developed in accordance with the 2022 Global Initiative for Chronic Obstructive Lung Disease (GOLD) guidelines. Between May 2022 and April 2023, 260 patients with COPD were prospectively enrolled from Beilun People’s Hospital in Ningbo, China. Patients received either mHealth-supported management (including electronic health record integration and regular remote guidance from healthcare providers) or usual care with routine discharge follow-up. The intervention duration was 12 months. Clinical outcomes, including pulmonary function parameters (FEV1, FEV1% predicted, FEF50, FEF75, MMEF), COPD Assessment Test (CAT), modified Medical Research Council (mMRC) dyspnea scale, and frequency of acute exacerbations, were compared between groups. All analyses were conducted under a modified intention-to-treat framework using the last observation carried forward (LOCF) method to account for missing data.

**Results:**

After 12 months, patients receiving mHealth-supported management demonstrated significant improvements in symptom burden and dyspnea, with CAT scores decreasing from 21.80 ± 3.24 to 17.30 ± 4.04 and mMRC scores from 2.43 ± 0.69 to 1.82 ± 0.75 (both *p* < 0.001). No significant overall improvement in FEV1 or FEV1% predicted was observed within groups, although between-group differences emerged at later time points. However, parameters reflecting small airway function improved significantly in the mHealth group, including FEF50, FEF75, and MMEF (all *p* < 0.001), whereas these measures declined in the usual care group. The annual frequency of acute exacerbations was significantly lower in the mHealth group (1.67 ± 0.80 vs. 2.24 ± 0.45, *p* < 0.001). In addition, the mHealth-based early warning system was associated with earlier healthcare utilization (mean reduction of 2.31 days), shorter hospital stays, and reduced medical costs.

**Conclusion:**

mHealth-supported COPD management was associated with improved symptoms, reduced dyspnea, enhanced small airway function, and fewer acute exacerbations in a real-world setting. Given the non-randomized design, these findings should be interpreted as reflecting associations rather than definitive causal effects. This approach may offer a practical strategy for improving long-term disease management and reducing healthcare burden in community-based COPD care.

## 1 Introduction

Chronic obstructive pulmonary disease (COPD) is a common, preventable, and treatable condition characterized by persistent respiratory symptoms and airflow limitation, which typically result from long-term exposure to harmful particles or gases (1). COPD has become a major global public health challenge. In 2019, it ranked as the third leading cause of death worldwide, accounting for more than 3.3 million deaths (2). In China, the estimated number of individuals with COPD among those aged ≥20 years exceeds 100 million, and the prevalence is projected to continue rising over the coming decades, imposing substantial clinical and economic burden (3).

Despite well-established diagnostic criteria, underdiagnosis and nonstandard diagnostic practices remain common, resulting in inappropriate treatment and suboptimal long-term management. Spirometry is essential for confirming the diagnosis; nevertheless, many patients present for their first clinical visit with already severely impaired lung function. In China, COPD prevalence is significantly higher in rural than in urban areas, and most patients initially seek care in primary healthcare settings (3). Acute exacerbations of COPD—episodes of acute symptom deterioration with increased risk of hospitalization—are a major driver of disease progression and a key contributor to the escalating healthcare burden (4). Therefore, the main goals of stable-phase management are to relieve symptoms and reduce the risk of future exacerbations. However, conventional COPD care has often emphasized reactive “rescue” treatment, with most patient–provider interactions occurring during exacerbation episodes and limited continuity of prevention-oriented management. These gaps highlight the need to shift toward an integrated, prevention-oriented model of COPD care.

The COVID-19 pandemic, first reported in 2019, disrupted healthcare systems worldwide and substantially affected routine chronic disease services, accelerating the adoption of innovative management strategies. Mobile health (mHealth) technology has gained increasing recognition as a patient-centered approach. By leveraging mobile devices (e.g., smartphones) and wearable sensors, mHealth can promote engagement and adherence through reminders, education, and remote monitoring, thereby improving self-management capacity (5, 6). Prior studies suggest that structured health coaching, particularly when initiated early after discharge, can improve quality of life and reduce readmission risk, and mHealth applications have been increasingly used to support self-management across chronic diseases (7, 8). In COPD, mHealth-enabled solutions facilitate longitudinal monitoring, medication reminders, and remote patient–provider interactions, and have been associated with improvements in self-management, exacerbation prevention, and psychological well-being. Widely used platforms such as WeChat may further enhance adherence by improving accessibility and interaction (9, 10). For example, Zhang et al. developed a smartphone-based Internet of Things system that supports medication reminders, data collection, health education, and patient–provider communication for COPD management (11). Mishra et al. (12). reported that early introduction of remote monitoring is an effective strategy to control COPD progression. Such digital systems may help maintain continuity of care during public health emergencies and provide a feasible pathway toward more integrated COPD management.

In this study, we implemented an integrated chronic disease management platform that supports individualized electronic records and enables prevention, screening, and early warning/management of acute exacerbations through health data collection, storage, and analysis. In parallel, we developed an mHealth-based COPD management system delivered via mHealth tools and the WeChat platform. Tertiary hospitals established dedicated disease-management teams and strengthened training for primary care physicians to support standardized implementation at the community level. The system was designed to be compatible with both Android and iOS devices, covering the vast majority of smartphone users, to align with prevailing usage patterns (13). Platform-generated data were used to support individualized optimization of treatment and to provide primary care physicians with timely, actionable information, thereby facilitating efficient diagnosis and management within a bidirectional referral framework. Regional data storage and sharing were enabled through the WeChat-based infrastructure. Through coordinated collaboration between tertiary hospitals and primary care institutions, this approach aimed to optimize a “hospital–community–home” continuum of care, reduce the occurrence of acute exacerbations, alleviate patient economic burden, and improve quality of life, thereby evaluating the feasibility and effectiveness of this integrated mHealth-enabled management model.

## 2 Subjects and methods

### 2.1 Study population

This prospective study was conducted at Beilun District People’s Hospital, Ningbo, China, between May 2022 and April 2023. Patients with chronic obstructive pulmonary disease (COPD) who had a history of hospitalization for acute exacerbation (AECOPD) were consecutively enrolled during routine clinical practice.

Eligible participants met the following inclusion criteria:

1. Diagnosis of COPD according to the 2022 Global Initiative for Chronic Obstructive Lung Disease (GOLD) criteria, classified as Group B or E;
2. Clinically stable condition at enrollment, with no acute exacerbation within the preceding 3 months;
3. Ability to perform self-care and comply with study procedures;
4. Ability to use a smartphone and operate the mobile application after training;
5. Willingness to use a provided pulse oximeter for monitoring oxygen saturation and heart rate before and after exercise.

Exclusion criteria included:

1. Severe comorbidities (e.g., coronary heart disease, severe hepatic or renal dysfunction, hematological disorders, severe heart failure, asthma, or malignancy);
2. Long-term systemic corticosteroid use or active infectious disease;
3. Requirement for mechanical ventilation during hospitalization;
4. Visual or hearing impairment affecting participation;
5. Lack of internet access at home.

All participants provided written informed consent prior to participation. The study protocol was approved by the institutional ethics committee. Participants received either an mHealth-supported management program or usual care follow-up, based on routine clinical management pathways. Usual care consisted of standard discharge follow-up according to existing clinical practice.

Patients received either mHealth-supported management or usual care based on routine clinical management pathways. Specifically, allocation was determined jointly by physicians according to clinical workflow and by patients’ willingness and ability to engage with the mHealth platform, rather than by random assignment. This design reflects real-world clinical practice.

### 2.2 Observation methods

**Figure 1:**
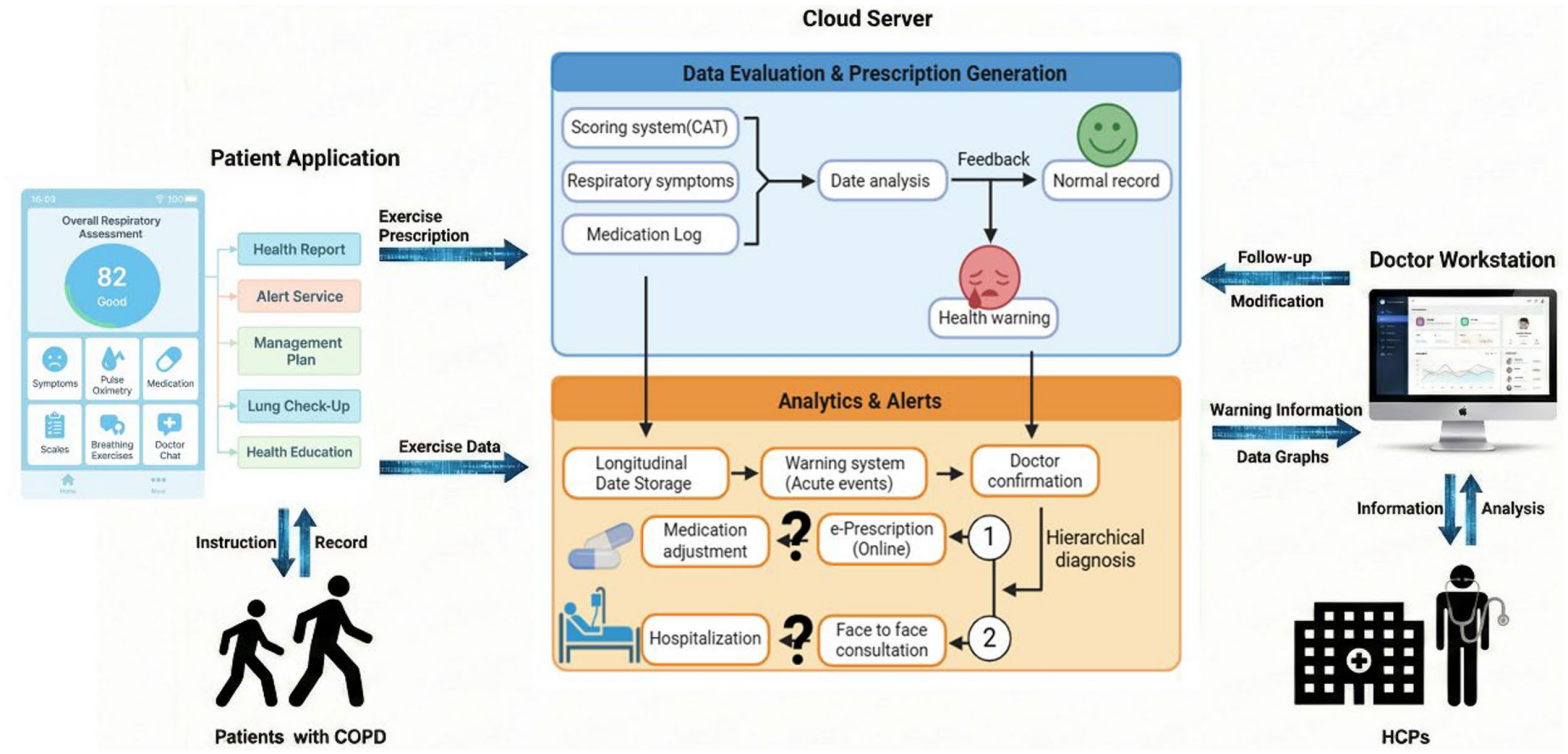
Schematic diagram of the mHealth-based closed-loop system architecture for remote monitoring and graded intervention of chronic respiratory diseases. The system comprises three core components: the patient application, cloud server, and doctor workstation. Patients use the mobile terminal to collect data including symptom scores/questionnaires (e.g., CAT), pulse oximetry, medication records, and respiratory training exercises, while receiving health reports, management plans, health education, and alert notifications. Simultaneously, they upload exercise performance data to the cloud. The cloud server performs longitudinal storage and trend analysis on multi-source follow-up data, automatically generating feedback and exercise prescriptions. When potential acute events or abnormal patterns are detected, alerts are triggered and pushed to the doctor workstation. The doctor workstation displays alert information and data charts. After confirming the alerts and completing graded diagnosis, physicians can conduct follow-up and adjust management plans (e.g., online electronic prescriptions/medication adjustment, recommending in-person consultation or further medical intervention). The intervention outcomes are then fed back into the system, forming a continuously iterative closed-loop management process.

In Figure 1, a WeChat-based mHealth COPD management system was employed in this study (14). This system was designed for long-term follow-up of patients with chronic respiratory diseases (represented by COPD), establishing a closed-loop management framework comprising the “patient application terminal—cloud platform—physician workstation” (Figure 1), enabling full-process management from data collection to intervention decision-making. The patient terminal serves as the entry point, where symptoms, blood oxygen levels, medication usage, and other information are recorded via the mobile application, and interventions such as respiratory training are implemented. Simultaneously, patients receive and provide feedback on health reports, alert notifications, and personalized exercise prescriptions generated by the cloud platform. The cloud platform functions as the core processing layer, integrating and analyzing multi-source data. When parameters remain stable, normal records are fed back; upon identifying abnormalities, health alerts are triggered. Leveraging longitudinal data storage and early warning models, alerts requiring physician confirmation are graded and pushed to support clinical judgment. The physician workstation presents trend charts and alert notifications of patients, assisting physicians in risk assessment and follow-up decision-making. Physicians can adjust prescriptions online, schedule in-person consultations, or initiate graded interventions such as referrals. These decisions are fed back into the system, forming a continuously optimized closed-loop management process.

In this study, enrolled patients engaged in self-management through the application, supervised by medical staff via the doctor workstation. The medical team comprised hospital physicians and general practitioners from community health centers, with clearly defined responsibilities: general practitioners assumed routine management duties, while hospital physicians were responsible for acute exacerbation treatment and specialized support. All management procedures strictly adhered to the established closed-loop pathway. Patients were required to submit daily data within specified time frames through the WeChat application, including medication adherence, blood oxygen monitoring (heart rate and oxygen saturation), smoking cessation status, symptom records, and respiratory rehabilitation exercises. Upon submission, data were uploaded in real-time to the terminal for analysis. Based on this information and individual patient circumstances, medical staff refined and personalized COPD treatment plans while promptly addressing alert notifications. This process encompassed diagnosis, initial assessment, initial management, review, and adjustment, thereby forming a comprehensive home-based prevention and management protocol for stable COPD patients that is grounded in integrated assessment and delivered through individually tailored, precisely implemented interventions.

Patients in the control group received usual care, which consisted of routine outpatient follow-up after discharge according to existing clinical practice. This included pharmacological treatment guided by GOLD recommendations, periodic outpatient visits, and standard health education provided during clinical encounters, but did not involve structured remote monitoring, daily symptom reporting, or real-time feedback through a digital platform.

### 2.3 Data collection and assessment

The effectiveness of the management program was evaluated using the following indicators: patient symptom scores, changes in pulmonary function before and after management, and frequency of acute exacerbations during the management period, as detailed in Supplementary Table S1.

#### 2.3.1 Pulmonary function assessment

Pulmonary function was measured for both groups using a spirometer at baseline and within one year of management. The assessment indicators included FEF50, FEF75, MMEF, FEV1, and FEV1% predicted, with the percentage of predicted FEV1 calculated accordingly. All FEV1 values reported hereafter refer to measurements obtained after bronchodilator inhalation.

#### 2.3.2 Acute exacerbation assessment

In this study, acute exacerbation of COPD was defined as follows: within a 14-day period, dyspnea, sputum purulence, and increased sputum volume were considered major symptoms (each scored 2 points), while rhinorrhea, nasal congestion, sore throat, cough, and wheezing were considered minor symptoms (each scored 1 point) (15). If the total symptom score exceeded 6 points for ≥2 consecutive days and exceeded the range of daily fluctuation, the system automatically detected a suspected exacerbation. This detection required further confirmation by healthcare providers to ensure that the symptoms were attributable to COPD. The time interval from the date of acute exacerbation identified by the APP to the date of the patient’s offline medical consultation was defined as the time to medical consultation after acute exacerbation. Prior to the study, the criteria were fully explained to patients in both groups to minimize missed diagnoses.

### 2.4 Statistical analysis

Statistical analysis was performed using R software (version 4.3.2; R Foundation for Statistical Computing, Vienna, Austria). Continuous variables are presented as mean ± standard deviation, and categorical variables as frequencies or percentages. Between-group comparisons were performed using the independent samples t-test or Mann–Whitney U test, as appropriate, while categorical variables were compared using the *χ*^2^ test. To minimize bias related to loss to follow-up, a modified intention-to-treat analysis was conducted, in which missing outcome data were handled using the last observation carried forward (LOCF) method. To assess the robustness of the findings, analyses based on complete-case data were also performed, and the results were compared with those from the LOCF-based analysis. A two-sided *p* value <0.05 was considered statistically significant.

## 3 Results

### 3.1 Patient enrollment and follow-up

**Figure 2:**
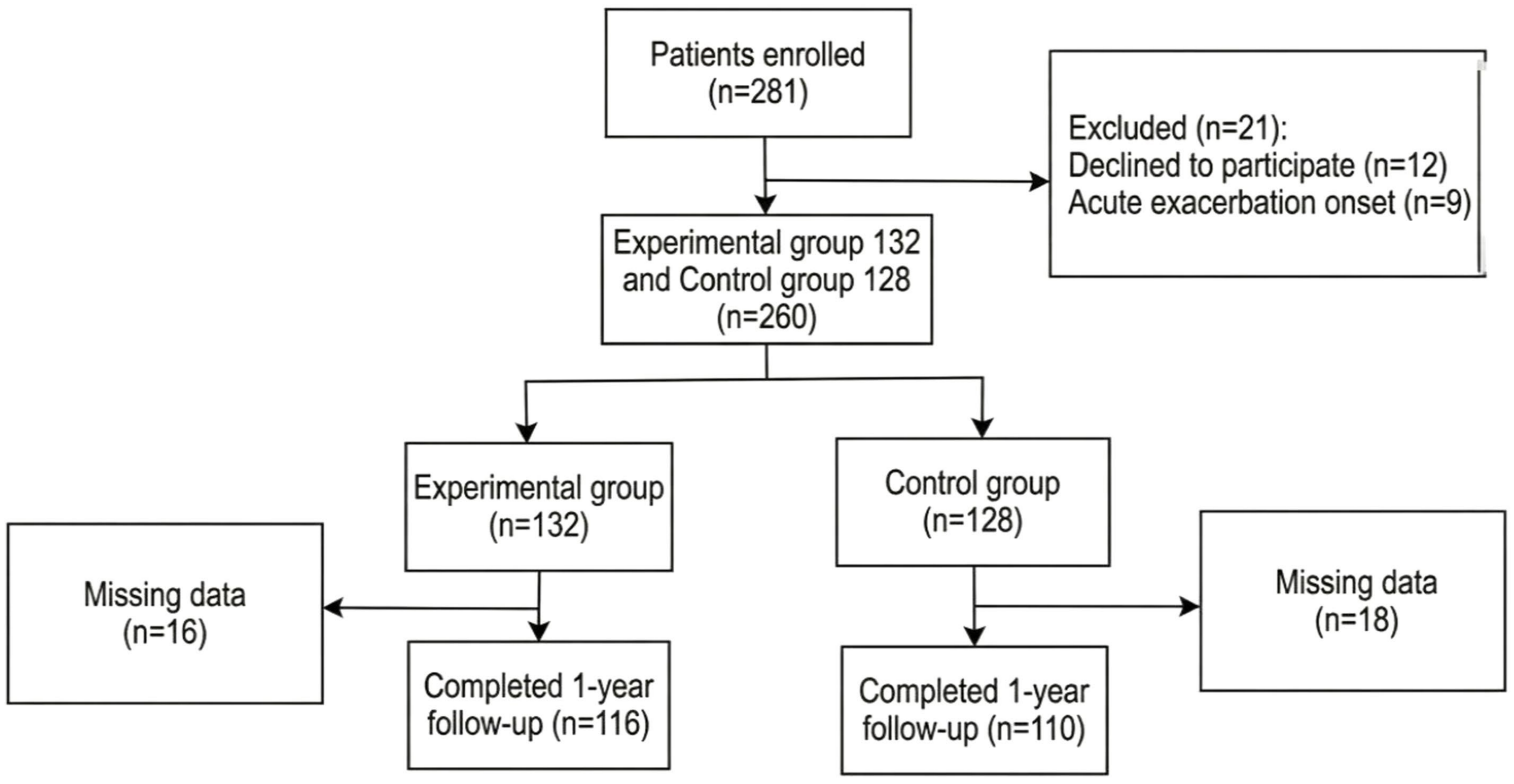
Flowchart of patient recruitment for the COPD study.

A total of 281 patients were initially screened for inclusion in this study. After excluding 21 patients who did not meet the eligibility criteria, 260 patients were included in the final analysis. Patients were allocated to either the mHealth-supported management group or the usual care group based on real-world clinical practice. In the mHealth group, 132 patients were enrolled, of whom 116 completed the study, with 16 patients lost to follow-up or withdrawn (attrition rate: 12.1%). In the usual care group, 128 patients were enrolled, and 110 completed the study, with 18 patients lost to follow-up or withdrawn (attrition rate: 14.1%). The detailed study flowchart and reasons for attrition are presented in Figure 2. Baseline characteristics of the two groups were comparable, and no statistically significant differences were observed in age, smoking history, lung function classification, or GOLD group (all *p* > 0.05). Detailed comparisons of baseline variables are presented in Table 1.

**Table 1:** Clinical baseline characteristics of patients with COPD.

Patient clinical characteristics	Experimental group (*n* = 132)	Control group (*n* = 128)	*p*
Gender			1.000
Male	92	90	
Female	40	38	
Age			0.923
≤40	7	8	
41–60	41	40	
>60	84	80	
Educational background			0.991
Junior high school and below	57	56	
High school or college degree	49	48	
University and above	26	24	
Smoking history			0.836
Former smoker	79	78	
Current smoker	16	17	
Non-smoker	37	33	
Smoking amount			0.961
≥20 packs-years	72	70	
<20 packs-years	60	58	
Lung function			0.986
FEV1% ≤ 50%	64	62	
FEV1% >50%	68	66	
COPD severity			0.963
Group B	60	58	
Group E	72	70	
Digital literacy			1.000
Good	64	62	
Medium	49	48	
Poor	19	18	
Medication use			1.000
Dual therapy (ICS + LABA)	50	48	
Triple therapy (ICS + LAMA + LABA)	82	80	

### 3.2 Clinical outcomes

All analyses were conducted under a modified intention-to-treat framework, in which missing outcome data were handled using the last observation carried forward (LOCF) method.

#### 3.2.1 Improvement in patient symptoms

The improvement in symptoms was assessed using CAT scores and mMRC dyspnea scores for both groups (Table 2). In the experimental group, the CAT score decreased significantly from 21.80 ± 3.24 before the intervention to 17.30 ± 4.04 after the intervention (*p* < 0.001), whereas no significant improvement in CAT score was observed in the control group (*p* > 0.05). Similarly, the mMRC score in the experimental group decreased significantly from 2.43 ± 0.69 before the intervention to 1.82 ± 0.75 after the intervention (*p* < 0.001); conversely, the control group showed no significant improvement in mMRC score (*p* > 0.05). The overall pattern of results was consistent with that observed in the complete-case analysis.

**Table 2:** Comparison of CAT and mMRC scores between the two groups.

CAT score				mMRC score			
Time	Experimental group (*n* = 132)	Control group (*n* = 128)	*p*-value	Time	Experimental group (*n* = 132)	Control group (*n* = 128)	*p*-value
Baseline	21.80 ± 3.24	21.52 ± 2.59	0.431	Baseline	2.43 ± 0.69	2.35 ± 0.63	0.396
3 months	20.42 ± 3.66	21.41 ± 3.88	0.039	3 months	2.22 ± 0.84	2.33 ± 0.68	0.171
6 months	18.11 ± 3.66	20.88 ± 4.08	<0.001	6 months	2.13 ± 0.79	2.32 ± 0.73	0.064
9 months	17.69 ± 3.90	20.60 ± 4.32	<0.001	9 months	2.02 ± 0.82	2.27 ± 0.73	0.016
12 months	17.30 ± 4.04	20.96 ± 2.26	<0.001	12 months	1.82 ± 0.75	2.20 ± 0.71	<0.001
*p*-value	<0.001	0.083		*p*-value	<0.001	0.088	

#### 3.2.2 Pulmonary function assessment

This study evaluated the improvement in pulmonary function following the management APP intervention from both large and small airway dimensions. Changes in pulmonary function are summarized in Table 3. At baseline, no significant differences were observed between the two groups in either absolute FEV1 or FEV1% predicted (both *p* > 0.05). Over the 12-month follow-up period, the mHealth group showed a gradual increase in absolute FEV1 (from 1.20 ± 0.26 L to 1.27 ± 0.31 L), whereas the control group demonstrated a slight decline (from 1.19 ± 0.28 L to 1.11 ± 0.35 L). Similar trends were observed for FEV1% predicted. Between-group differences became statistically significant from 6 months onward for both absolute FEV1 and FEV1% predicted (all *p* < 0.001). However, the within-group longitudinal change in FEV1 did not reach statistical significance in either group (*p* = 0.0571 and *p* = 0.0555, respectively). These findings suggest that the mHealth intervention may help maintain lung function over time rather than produce a marked improvement.

**Table 3:** Comparison of FEV1 between the two groups.

Time	Absolute FEV1 (L)			FEV1% predicted		
	Experimental group (*n* = 132)	Control group (*n* = 128)	*p*-value	Experimental group (*n* = 132)	Control group (*n* = 128)	*p*-value
Baseline	1.20 ± 0.26	1.19 ± 0.28	0.750	52.28 ± 6.04	52.06 ± 7.14	0.788
3 months	1.24 ± 0.27	1.17 ± 0.30	0.060	53.09 ± 6.77	51.15 ± 8.15	0.043
6 months	1.26 ± 0.30	1.11 ± 0.30	<0.001	53.03 ± 7.28	49.37 ± 8.90	<0.001
9 months	1.26 ± 0.30	1.10 ± 0.33	<0.001	53.69 ± 7.74	49.81 ± 8.72	<0.001
12 months	1.27 ± 0.31	1.11 ± 0.35	<0.001	53.06 ± 8.10	49.08 ± 9.10	<0.001
*p*-value	0.0571	0.0555		0.396	0.005	

Furthermore, regarding small airway function, as shown in Table 4. At baseline, no significant differences were observed between the two groups in FEF50, FEF75, or MMEF (all *p* > 0.05). During follow-up, the mHealth group showed a progressive improvement in all three parameters. FEF50 increased from 47.39 ± 3.84 to 54.23 ± 4.75, FEF75 from 43.71 ± 4.02 to 50.20 ± 5.32, and MMEF from 55.61 ± 5.37 to 63.66 ± 8.43 over 12 months (all *p* < 0.001 for within-group comparisons). In contrast, the control group demonstrated a gradual decline or minimal change over time. Between-group differences became statistically significant from 6 months onward for FEF50 and from 3 months onward for FEF75 and MMEF, and remained significant throughout the follow-up period (all *p* < 0.001 at later time points). These findings suggest a sustained improvement in small airway function in the mHealth group compared with usual care.

**Table 4:** Comparison of small airway function between the two groups.

Time	FEF50			FEF75			MMEF		
	Experimental group (*n* = 132)	Control group (*n* = 128)	*p*-value	Experimental group (*n* = 132)	Control group (*n* = 128)	*p*-value	Experimental group (*n* = 132)	Control group (*n* = 128)	*p*-value
Baseline	47.39 ± 3.84	48.33 ± 4.58	0.075	43.71 ± 4.02	44.05 ± 4.34	0.513	55.61 ± 5.37	54.43 ± 5.46	0.082
3 months	49.25 ± 4.27	48.60 ± 4.98	0.273	44.92 ± 4.41	42.96 ± 4.54	0.003	57.93 ± 5.77	53.53 ± 5.85	<0.001
6 months	50.29 ± 4.45	47.66 ± 4.95	<0.001	45.40 ± 4.96	42.35 ± 4.87	<0.001	60.05 ± 6.91	53.03 ± 6.32	<0.001
9 months	52.79 ± 4.66	47.67 ± 5.20	<0.001	49.04 ± 4.93	42.03 ± 5.16	<0.001	62.37 ± 7.55	52.55 ± 7.27	<0.001
12 months	54.23 ± 4.75	46.39 ± 5.80	<0.001	50.20 ± 5.32	41.07 ± 5.69	<0.001	63.66 ± 8.43	52.33 ± 7.62	<0.001
*p*-value	<0.001	0.005		<0.001	<0.001		<0.001	0.017	

The overall results were consistent with those obtained from the complete-case analysis.

#### 3.2.3 Assessment of acute exacerbation-related indicators

Analysis of mHealth cloud data over the one-year follow-up period (Table 5) showed that the mean frequency of acute exacerbations was 1.67 ± 0.80 per patient in the experimental group (n = 116) and 2.24 ± 0.45 in the control group (n = 110), with a significantly lower rate observed in the experimental group (*p* < 0.05). As this analysis was based on complete follow-up data over 12 months, only patients who completed the full observation period were included.

**Table 5:** Comparison of acute exacerbation frequency between the two groups.

Time	Experimental group (*n* = 116)	Control group (*n* = 110)	*p*-value
Baseline (1 year before enrollment)	2.14 ± 0.66	2.23 ± 0.48	0.248
During the past year	1.67 ± 0.80	2.24 ± 0.45	<0.001
*p*-value	<0.001	0.82	

Furthermore, the mHealth system appeared to facilitate earlier recognition of exacerbations. Patients in the experimental group sought medical consultation approximately 2.31 days earlier than those in the control group, which was associated with shorter hospital stays and lower medical costs (Supplementary Table S2).

One patient in the control group required ICU admission, and no deaths were recorded in either group during the study period.

#### 3.2.4 Assessment of patient adherence

**Figure 3:**
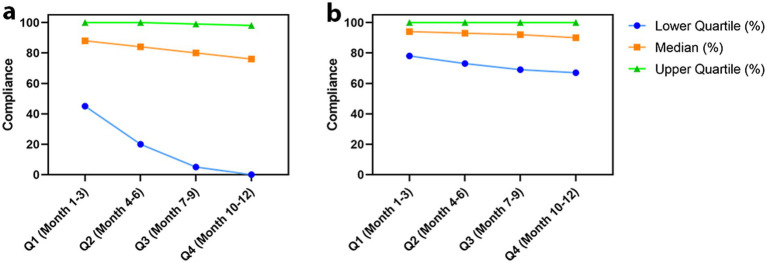
Trends in quarterly adherence during the one-year follow-up period. **(a)** All enrolled patients (*n* = 132): The lower quartile (Q1) declined over time, indicating an increasing proportion of patients with low or missing adherence. The median showed a slight decrease, while the upper quartile (Q3) remained high. **(b)** Patients completing one-year follow-up (*n* = 116): Adherence remained consistently high across all quarters, with the median around 90% and Q3 approaching 100%. Missing data may reflect either loss to follow-up or intermittent non-reporting; therefore, adherence interpretation focuses on patients completing follow-up to reduce attrition-related bias.

This study analyzed patient adherence to mHealth-based management. The results revealed that adherence to the system did not exhibit uniform decay over time but rather demonstrated a pronounced bipolar trend. Among all enrolled patients (Figure 3a), approximately 25% of low-adherence patients showed continuously declining adherence levels as follow-up progressed, approaching near-zero adherence in the later stages; meanwhile, approximately 25% of high-adherence patients maintained adherence rates close to 100% throughout the long-term follow-up. This stratification pattern is consistent with the “usage decay” phenomenon commonly observed in mobile health interventions, highlighting the challenge of behavioral maintenance during extended follow-up periods. Further analysis suggested that data missingness in the later-stage low-adherence group primarily resulted from behavioral factors (e.g., withdrawal, discontinuation of use). However, in the subgroup that completed the full follow-up (Figure 3b), although overall adherence was relatively high, a mild decline was still observed at the lower percentile, which more likely reflects the combined effect of technical non-adherence and mild adherence fatigue.

In the adherence analysis, this study specifically focused on the correctness of inhaler device usage. Regarding patients who used the device correctly, both groups collected data only from those who attended all four follow-up visits, through 12 months of combined online and offline training on correct inhalation medication administration provided to the experimental group, the correct usage rate increased from 46/116 (39.7%) to 106/116 (91.4%). In contrast, the correct usage rate in the control group increased from 40/110 (36.4%) to 80/110 (72.7%). After 12 months of repeated guidance on inhaler medication use through the mHealth software, a statistically significant difference in correct usage rates emerged between the two groups (Supplementary Table S3).

## 4 Discussion

With the accelerating population aging in China, the disease burden of chronic obstructive pulmonary disease (COPD) is becoming increasingly substantial. In 2024, the National Health Commission officially incorporated COPD into the National Basic Public Health Service Program, marking a new stage in its prevention and control. Primary care communities represent both a major focus of COPD management and a weak link in the current healthcare system, and strengthening the capacity of community healthcare workers is critical to achieving systematic community-based management (16). The traditional hospital-centered, passive and reactive model has significant limitations: patients’ health status is assessed only during brief outpatient follow-up visits, whereas during the long intervals outside the hospital, often lasting several months, fluctuations in disease status, medication-related problems, and early signs of acute exacerbation are frequently overlooked, creating a “management vacuum” in which patients are effectively left without adequate supervision. Therefore, to address this challenge and overcome the temporal and spatial limitations of conventional care, there is an urgent need to develop a new information-based and proactive management model capable of continuous and seamless monitoring and management. This is essential not only for improving the quality of care, but also for implementing national public health policy and safeguarding the long-term health of patients.

The core value of the mHealth software developed in this study lies in the establishment of an integrated collaborative management platform that goes beyond simple data recording by deeply incorporating clinical pathways and intelligent algorithms (17). Its main strengths are reflected in two closed-loop processes. First, it establishes an integrated intelligent management loop on the patient side. The mHealth system integrates multidimensional data, including symptom assessments (mMRC/CAT), physiological parameters, medication use, and pulmonary rehabilitation training, and performs real-time risk analysis using embedded algorithms. Once early warning signals of acute exacerbation are identified, such as a sharp increase in CAT score, the system immediately delivers personalized response recommendations to patients and sends alerts to healthcare providers, thereby enabling proactive management and effectively bridging the gap in out-of-hospital care. Second, it creates a physician–patient collaborative decision-support loop. The mHealth system also serves as an efficient clinical tool by integrating fragmented out-of-hospital data into visualized charts and structured reports, allowing physicians to rapidly understand disease trajectories and improve decision-making efficiency. At the same time, the built-in secure communication channel supports non-urgent text-and-image consultations, fostering a more equal and collaborative physician–patient relationship and truly empowering the long-term, systematic management of chronic disease.

In this 12-month hospital–community intervention study, we evaluated the effectiveness of a closed-loop management model based on the mHealth system for patients with chronic obstructive pulmonary disease (COPD).

Importantly, the main findings remained consistent when analyzed under a modified intention-to-treat framework using the LOCF method, and similar trends were observed in complete-case analyses, supporting the robustness of the results.

First, with respect to symptoms and quality of life, the mHealth intervention produced rapid and clear improvements. Through continuous self-monitoring, educational feedback, and physician–patient communication, the CAT score in the intervention group decreased significantly from 21.80 ± 3.24 at baseline to 17.30 ± 4.04, representing a reduction of approximately 20.6%. Meanwhile, the mMRC score decreased from 2.4 ± 0.7 to 1.8 ± 0.8, indicating an improvement in dyspnea severity. These findings are highly consistent with the GOLD guidelines and previous COPD studies, which have shown that standardized self-management and pulmonary rehabilitation can effectively alleviate symptoms and improve health-related quality of life (HRQoL) (18).

Second, the effects of mHealth on pulmonary function were evaluated separately in terms of large and small airway indices. Forced expiratory volume in 1 s (FEV1), the traditional key indicator of large-airway obstruction, did not show a statistically significant change over 12 months, which is consistent with most comparable studies (16, 17). However, between-group differences became evident at later time points, suggesting that the mHealth intervention may help maintain lung function rather than produce marked improvement. In contrast, the indicators reflecting small-airway function showed a distinctly different and positive pattern. In the intervention group, FEF50, FEF75, and MMEF increased by 14.4, 14.8, and 14.5%, respectively, whereas these parameters declined by 4–7% in the control group over the same period. Small airways are the principal site of early pathological changes in COPD. These findings suggest that the continuous respiratory rehabilitation training, real-time medication supervision, and symptom warning loop delivered through the mHealth system may contribute to improvements in small-airway function, although the underlying mechanisms cannot be directly determined from this study. Although a significant improvement in FEV1 may require longer observation, the early recovery of small-airway mechanics may provide an important foundation for slowing the accelerated age-related decline in FEV1.

Finally, the mHealth intervention demonstrated particularly notable benefits in clinically important outcomes and healthcare economic burden. In the intervention group, the annual mean number of acute exacerbations decreased from 2.14 to 1.67 episodes. More importantly, the healthcare resource consumption associated with each exacerbation was also effectively reduced. Compared with conventional management, mHealth intervention shortened the average length of hospitalization by 4.0 days per patient and reduced the corresponding direct medical costs by 26%. As this analysis was based on patients who completed the full 12-month follow-up, the results should be interpreted with some caution, although the overall findings were consistent with the main analysis. These findings not only substantially reduced the economic and psychosocial burden on patients, but also support previous international studies and real-world studies from Shanghai, China, showing that intensified community-based management can reduce healthcare utilization related to acute exacerbations (19, 20). Collectively, these results highlight the considerable potential of this management model to optimize overall healthcare resource utilization and reduce the societal burden of disease.

In real-world COPD management, the central challenge of inhalation therapy lies in translating prescription into correct technical execution. At the beginning of this study, only 39.7% of patients were able to use the Symbicort inhaler correctly, directly confirming observations in the literature that although the efficacy of inhaled therapy has been established in clinical trials (21, 22), its effectiveness in routine practice is often substantially attenuated by poor adherence and incorrect inhaler technique (23, 24). Through 1 year of structured community-based supervision, the correct-use rate increased to 91.4%, and a significant reduction in the frequency of acute exacerbations was observed in the intervention group. These findings demonstrate that systematic long-term reinforcement of inhaler technique can effectively bridge the gap between efficacy observed in clinical trials and effectiveness achieved in routine practice, thereby ensuring that the biological effects of inhaled therapy are translated into real clinical benefit. This also underscores the importance of targeting medication-use technique as a core component of chronic disease management.

During the study, system-derived data also showed that patients maintained relatively high adherence throughout the management period, which may have been one of the key factors underlying the positive effects of the intervention. This may be attributable to the combined contribution of three factors. First, the mHealth system provided a technical foundation for personalized adjustment of tasks and rules, and its short-cycle feedback mechanism facilitated the establishment of physician–patient trust and enabled dynamic responses to treatment plans, thereby sustaining patient engagement. Second, the WeChat platform served as a convenient communication channel and mutual-support community. Group-based interactions not only enabled prompt resolution of problems and sharing of self-management skills, but also provided social support that strengthened patients’ acceptance of long-term management.

Despite these encouraging findings, several limitations should be acknowledged. First, this was a non-randomized real-world study, and group allocation was based on routine clinical pathways and patient willingness to engage with the mHealth system. Therefore, we explicitly acknowledge that potential confounding factors, including patient motivation, digital literacy, and social support, may have influenced both adherence and clinical outcomes. Second, although baseline characteristics were comparable, residual confounding due to unmeasured variables cannot be fully excluded. Third, the study was conducted in a single center and required participants to have access to smartphones and internet connectivity, which may limit the generalizability of the findings to populations with lower digital literacy or fewer resources. Finally, the sample size was relatively limited, and no formal sample size calculation was performed prior to the study.

In summary, community-based closed-loop management supported by mHealth is not merely a tool, but a systematic strategy capable of integrating patient education, rehabilitation, monitoring, and early warning. However, the findings of this study should be interpreted as associative rather than causal, and further randomized and multicenter studies are warranted to confirm these results.

## Data Availability

The original contributions presented in the study are included in the article/Supplementary material, further inquiries can be directed to the corresponding authors.
