# Supplementary material for: Mobile health (mHealth)-supported community management of COPD: a prospective real-world study on clinical outcomes and exacerbation reduction
Source: Front Public Health. 2026 May 7;14:1836687. doi: 10.3389/fpubh.2026.1836687 (PMC13190599; doi:10.3389/fpubh.2026.1836687)
Supplement: Supplementary file 1 [file Data_Sheet_1.DOCX]

**Table S1. Data Collection Indicators and Sources**

| Data | Project | Collecting Time | Source of Data |
| --- | --- | --- | --- |
| Main clinical indicators | CAT score | At baseline and within 1 year of follow-up | APP data |
|  | Pulmonary function | At baseline and within 1 year of follow-up | Pulmonary function instrument |
|  | Number of acute attacks | Within 1 year of follow-up | APP data + in-hospital His system |
| Secondary clinical indicators | Time to Seek Medical Attention after Acute Attack | Within 1 year of follow-up | Time difference from App alert to offline visit |
|  | Number of Outpatient and Inpatient Visits | Within 1 year of follow-up | In-hospital His system |
|  | Number of Hospitalizations | Within 1 year of follow-up | In-hospital His system |
|  | Hospitalization Costs | Within 1 year of follow-up | In-hospital His system |
| Adverse Events | Death | Within 1 year of follow-up | In-hospital His system |

**Table S2. Comparison of timeliness of medical consultation after acute exacerbation and hospitalization costs between the two groups**

|  | Experimental Group（n=116） | Control Group（n=110） | P-value |
| --- | --- | --- | --- |
| Visit time (days) | 4.93±1.81 | 7.24±2.40 | <0.001 |
| Length of hospital stay (days) | 10.35±2.61 | 14.27±3.07 | <0.001 |
| Medical costs (RMB) | 9904.26±5632.37 | 13537.16±6835.38 | <0.001 |
| ICU admission (%) | 0 | 1% |  |
| Mortality rate (%) | 0 | 0 |  |

**Table S3. Comparison of the number of patients with correct use of inhaled medications between the two groups**

|  | Completely correct | | Partially correct | | Incorrect | |  |
| --- | --- | --- | --- | --- | --- | --- | --- |
| Time | Experimental Group（n=116） | Control Group（n=110） | Experimental Group（n=116） | Control Group（n=110） | Experimental Group（n=116） | Control Group（n=110） | P |
| Baseline | 46 | 40 | 58 | 55 | 12 | 15 | 0.714 |
| 3months | 60 | 52 | 46 | 45 | 10 | 13 | 0.665 |
| 6months | 72 | 60 | 36 | 40 | 8 | 10 | 0.505 |
| 9months | 87 | 70 | 25 | 34 | 4 | 6 | 0.178 |
| 12months | 106 | 80 | 10 | 28 | 0 | 2 | <0.001 |
